# Medical School Scholarships

**Published:** 1910-09-10

**Authors:** 


### Medical School Scholarships.

The efforts which were made not long ago to centralise the teaching of the non-clinical medical sciences in London were, as most members of the profession know, only partially successful. With no desire on the present occasion to reanimate the smouldering fires of the controversy to which those efforts gave rise, it is at least permissible to point out one way in which the arrangement finally effected has led, and still leads, to economy of time, money, and labour; and to show that in this especial respect the participation of the schools which held aloof from the concentration movement would produce benefits to themselves without any attached disadvantage. We allude to the conduct of hospital entrance scholarship examinations, in connection with which the waste of energy at present is nothing less than sinful. Last week a notice appeared among the London University announcements to the effect that the London Inter-Collegiate Scholarships Board will hold a combined entrance examination for twenty-three medical entrance scholarships, tenable at University College, University College Hospital Medical School, King's College, King's College Hospital, St. George's Hospital, Westminster Hospital, and the London School of Medicine for Women.

The immense saving of money which is effected by this scheme needs no explanation. Be it observed, however, that it is not merely the labours of the examinees that are economised; the examiners also are spared a great amount of trouble and possible inconvenience, and the supervision known technically as invigilating is also greatly reduced; a form of labour than which there are few things more tedious and less rewarded. If any testimonial as to the value of this system of joint examinations is needed it is only necessary to observe what has happened at Oxford and Cambridge. Up to about twenty years ago each college held a separate scholarship examination, a method as utterly prodigal and expensive to all concerned as the present habit of the London medical schools. Gradually the smaller colleges were compelled to band themselves together into " groups " of three, four, or five for these purposes; and the sequel of this movement is instructive. The largest colleges at first held aloof, knowing (or at least believing) that they were sure of the pick of the market. But in a few years it became evident that in self-defence they also would be compelled to admit the same principle, for the attractions of a group examination, at which perhaps forty or fifty scholarships were offered, proved often superior to the *prestige* even of such seminaries as Trinity or Balliol. It is to be understood that the colleges retain their separate identity in every other matter; therefore, surely even those schools whose authorities are most insistent on continued individuality need not fear the results of combined scholarship examination. Nay, if they distrust London University so much, why should they not amalgamate into another group? We can see no reason against it, and have given some excellent reasons for it. If the experience of the older universities is any guide, as it certainly must be, the measure will be imperative presently in sheer self-defence. Why not anticipate this by a timely reform which is to everybody's advantage?

